# Artificial Intelligence-Assisted Remote Monitoring in Children with Mixed Dentition: Agreement with Clinical Assessment in a Prospective Pilot Study

**DOI:** 10.3390/healthcare14213689

**Published:** 2026-10-31

**Authors:** Sara Caruso, Arianna Viarchi, Antonella Mattei, Emanuela Lisciotto, Vincenzo D’Antò, Marco Severino, Michele Tepedino, Roberto Gatto

**Affiliations:** 1 Department MeSVA, University of L’Aquila, 67100 L’Aquila, Italy; sara.caruso@student.univaq.it (S.C.); arianna.viarchi@student.univaq.it (A.V.); antonella.mattei@univaq.it (A.M.); michele.tepedino@univaq.it (M.T.); roberto.gatto@univaq.it (R.G.); 2 Department of Neuroscience, Reproductive Sciences and Oral Sciences, Section of Orthodontics, University of Naples Federico II, 80131 Naples, Italy; vincenzo.danto@unina.it; 3 Department of Medicine and Surgery, University of Perugia, Severi Square n.1, 06132 Perugia, Italy; marco.severino@unipg.it

**Keywords:** artificial intelligence, teledentistry, remote monitoring, pediatric dentistry, mixed dentition, oral hygiene, DentalMonitoring, digital dentistry

## Abstract

**Background**: Artificial intelligence (AI)-assisted remote monitoring is increasingly used in dentistry, but evidence in children who are not undergoing active orthodontic treatment remains limited. This prospective pilot study evaluated the agreement between an AI-based Observational Monitoring system and conventional clinical examination for selected oral health findings in children with mixed dentition. **Methods**: Twenty children (10 males and 10 females; mean age 10.5 years, range 9–12 years) were evaluated at baseline (T0), 6 months (T1), and 12 months (T2). Six clinical conditions—dental plaque, persistent dental calculus, minor food debris, mild gingivitis, satisfactory oral hygiene, and dental staining—were assessed by a clinical observer and by the DentalMonitoring Observational Monitoring system. None of the patients included in the study were undergoing orthodontic treatment. Observer and AI classifications were analyzed as paired data. For each condition and time point, observed agreement and Cohen’s kappa were calculated from the paired 2 × 2 table, and systematic differences between methods were assessed with the exact McNemar test; changes in prevalence over time were assessed with Cochran’s Q test. **Results**: Observed agreement ranged from 65% to 95% at T0, from 65% to 100% at T1, and from 85% to 100% at T2, with wide 95% confidence intervals (e.g., 65.0%, 95% CI 40.8–84.6%). Cohen’s kappa ranged from −0.14 to 0.64 and was not computable when a condition was absent according to both methods. For low-prevalence findings, high observed agreement coincided with kappa values close to zero. For conditions with more balanced prevalence, chance-corrected agreement was only fair. During follow-up, the prevalence of dental plaque and food debris decreased, whereas that of satisfactory oral hygiene and dental staining increased. **Conclusions**: AI-assisted Observational Monitoring showed moderate-to-high observed agreement but variable chance-corrected agreement with direct clinical assessment in this small pediatric cohort. The system may represent a complementary tool for longitudinal dental follow-up, but professional supervision remains necessary and larger validation studies are required.

## 1. Introduction

Artificial intelligence (AI) is increasingly being integrated into dentistry to support image interpretation, diagnosis, treatment planning, and clinical decision-making [1,2]. In orthodontics and teledentistry, AI-assisted remote monitoring systems allow standardized intraoral images to be collected outside the dental office and evaluated longitudinally, thereby complementing conventional chairside examinations and potentially enabling more continuous clinician-supervised follow-up [3,4,5].

DentalMonitoring version 7.54.0 (Dental Monitoring SAS, Paris, France) is an AI-assisted remote monitoring platform that combines a smartphone application, a dedicated image-acquisition device, and automated image-processing algorithms. Patients or caregivers periodically acquire standardized intraoral image sequences at home, while the software evaluates predefined clinical parameters that can subsequently be reviewed by the clinician. Previous studies have investigated remote monitoring for orthodontic tooth movement, aligner tracking, oral hygiene, and treatment follow-up [3,6,7,8,9,10].

Despite these promising applications, systematic reviews have reported substantial methodological heterogeneity and limited certainty of evidence for several teledentistry and remote-monitoring outcomes [11,12]. Moreover, most available evidence concerns adolescents or adults undergoing active orthodontic treatment, whereas data in younger children observed during dental development remain limited. The Observational Monitoring workflow may be particularly relevant in this setting because it allows children who do not currently require active treatment to be followed remotely through periodic intraoral image acquisition. During mixed dentition, such longitudinal observation may complement conventional examinations and help identify changes that warrant an in-office reassessment.

Accordingly, the aim of this prospective pilot study was to compare assessments generated by the DentalMonitoring Observational Monitoring system with those obtained during conventional clinical examination in children with mixed dentition. Six oral health findings were evaluated at baseline and during a 12-month follow-up: dental plaque, persistent dental calculus, minor food debris, mild gingivitis, satisfactory oral hygiene, and dental staining.

## 2. Materials and Methods

### 2.1. Study Design and Participants

This prospective observational pilot study included 20 pediatric patients, equally distributed by sex (10 males and 10 females), with a mean age of 10.5 years (range: 9–12 years). As a pilot investigation, the sample size was not derived from a formal a priori power calculation but was chosen on the basis of feasibility, reflecting the number of eligible patients who could be reliably enrolled and followed for the full 12-month period within the available study timeframe. This preliminary sample was considered adequate to obtain initial, exploratory estimates of observer–AI agreement and to inform the design and sample-size calculation of future, adequately powered confirmatory studies [13].

Inclusion criteria were: presence of mixed dentition; absence of a need for orthodontic treatment during the monitoring period; eligibility for remote observational follow-up, including availability of a smartphone compatible with the DentalMonitoring application and willingness of a parent or caregiver to assist with home image acquisition; and written informed consent provided by a parent or legal guardian.

Exclusion criteria were: systemic diseases with potential effects on oral or periodontal health; ongoing pharmacological treatment known to affect the periodontal or oral soft-tissue status; need for orthodontic treatment during the monitoring period; and inability of the parent or caregiver to comply with the home image-acquisition protocol. None of the patients included in the study were undergoing orthodontic treatment or required orthodontic treatment during the study period. Beyond these criteria, general health status, presence of systemic or oral pathologies, and current medication use were specifically reviewed for all candidates at enrollment; no participant included in the final sample presented relevant systemic or oral comorbidities, or was taking medications with known effects on periodontal or soft-tissue status, other than the six oral conditions specifically investigated in this study.

Participants were evaluated at three time points: baseline (T0), 6 months after baseline (T1), and 12 months after baseline (T2). At baseline, all children and their caregivers received standardized oral hygiene instructions; professional oral hygiene was performed at T0 and T1; no additional preventive interventions (e.g., fluoride varnish, sealants) or dietary counselling were provided; any restorative treatment performed during follow-up was recorded. These interventions were not part of the study protocol but may have influenced the prevalence of the investigated conditions over time.

### 2.2. Clinical Assessment

At each study visit, a trained clinical observer performed a standardized intraoral examination to assess the patient’s general oral health status. All clinical examinations were performed by the same single examiner, an orthodontic specialist with several years of clinical experience in pediatric dentistry and orthodontics. Prior to the start of the study, the examiner underwent a calibration exercise under the supervision of a senior orthodontist (co-author), during which the clinical criteria for each of the six investigated conditions were reviewed and standardized using reference clinical photographs and a pilot subset of cases not included in the final analysis, until full agreement on the classification criteria was reached. The following six predefined conditions were recorded as present or absent: small amount of dental plaque, persistent dental calculus, minor food debris, mild gingivitis, satisfactory oral hygiene, and dental staining. The same clinical conditions were independently assessed using the AI-assisted Observational Monitoring workflow. The clinical examiner was blinded to the AI-generated results, which were retrieved from the platform only after the clinical examination had been recorded.

For each condition, a binary present/absent classification was used, based on simplified criteria derived from established periodontal and hygiene indices, adapted to match the qualitative output categories generated by the DentalMonitoring Observational Monitoring system. Dental plaque was recorded as present when visible plaque accumulation was detectable on the dental surfaces, consistent with the criteria of the Plaque Index [14]. Persistent dental calculus and minor food debris were assessed according to the debris and calculus components of the Simplified Oral Hygiene Index (OHI-S) [15]. Mild gingivitis was recorded as present in cases of visible marginal gingival inflammation, following the criteria of the Gingival Index [14]. Satisfactory oral hygiene was defined as the absence of clinically detectable plaque, calculus, or food debris on the examined surfaces, in accordance with the same reference indices [14,15]. Dental staining was assessed according to a simplified adaptation of the Lobene Stain Index, considering the presence or absence of visible extrinsic tooth discoloration [16].

### 2.3. AI-Assisted Remote Monitoring

During follow-up, participants were enrolled in the Observational Monitoring protocol of the DentalMonitoring platform version 7.54.0 (Dental Monitoring SAS, Paris, France, version 7.54.0). Intraoral image sequences were acquired using a smartphone, the DentalMonitoring application, and the ScanBox positioning device. Scans were performed at home by the patient under the supervision or with the assistance of a parent or caregiver, according to instructions provided by the clinician. The Observational Monitoring module of the DentalMonitoring application was used, with the same software and algorithm version at T0, T1 and T2 throughout the 12-month study. The exact interval between home image acquisition and clinical examination at each time point was not systematically recorded.

The acquired images were processed through the DentalMonitoring platform, which generated AI-based assessments of the predefined oral conditions. AI-generated findings obtained at T0, T1, and T2 were compared with the corresponding clinical assessments. For each scan, the platform automatically reported the presence or absence of each predefined finding; no confidence scores or adjustable thresholds were available to the user. Image sequences were accepted only if they passed the platform’s automatic quality check; two image sequences were excluded because of insufficient image quality. AI-generated outputs were recorded as provided by the system, without being reviewed, modified or confirmed by a clinician before inclusion in the analysis.

### 2.4. Data Protection and Informed Consent

Written informed consent was obtained from the parents or legal guardians of all participants before enrollment. In addition, the written assent of the children was obtained using age-appropriate information. Parents or guardians were informed about the study procedures and about the acquisition, transfer, and processing of intraoral images through the DentalMonitoring platform. Personal and clinical data were handled in accordance with Regulation (EU) 2016/679 (General Data Protection Regulation, GDPR). Clinical interpretation and patient management remained under the responsibility of the treating clinician.

### 2.5. Ethical Approval

The study protocol was approved by the Institutional Review Board of the University of L’Aquila for studies involving humans (document DR206/2013) (approval code: 41/2022, approval date: 27 September 2022). The study was conducted in accordance with the principles of the Declaration of Helsinki and its subsequent amendments. DR206/2013 refers to the Rectoral Decree regulating the Institutional Review Board of the University of L’Aquila; the present study protocol was specifically approved with code 41/2022 on 27 September 2022 and covered home image acquisition, transfer of intraoral images to the DentalMonitoring platform and their AI-based processing.

### 2.6. Statistical Analysis

A descriptive analysis of all variables was performed using absolute frequencies and percentages. Because each child was assessed both by the clinical observer and by the AI-based remote monitoring system, observer and AI classifications were treated as paired data. At each time point (T0, T1, and T2), differences between the diagnoses made by the observer and those obtained with the AI system were evaluated with the exact McNemar test. For each dichotomous variable and time point, agreement between the observer and the AI system was assessed with Cohen’s kappa coefficient. Observed percentage agreement was also calculated from the paired 2 × 2 table as (a + d)/n, with n = 20, and its 95% confidence interval (CI). Finally, changes in each variable across the three time points (T0, T1, and T2) were evaluated separately for the observer and for the AI system using Cochran’s Q test. Data were analyzed using STATA/BE version 18.0 (StataCorp LLC, College Station, TX, USA). All statistical tests were two-sided, and a *p*-value < 0.05 was the criterion for statistical significance.

## 3. Results

**Figure 1:**
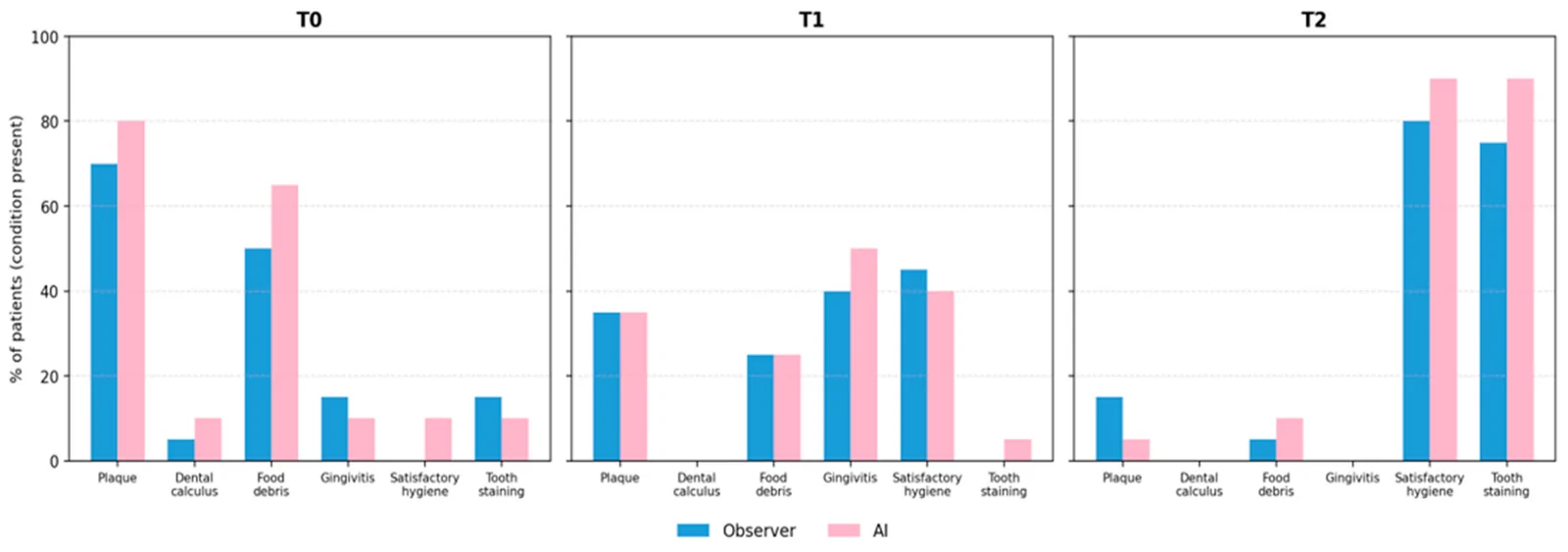
Percentage of the six oral-health indicators (dental plaque, persistent dental calculus, minor food debris, mild gingivitis, satisfactory oral hygiene, and dental staining) recorded by the clinical observer and the AI system at T0, T1, and T2.

The study sample consisted of 20 children (10 males and 10 females) with a mean age of 10.5 years (range 9–12 years), all in the mixed dentition and none undergoing orthodontic treatment. All participants completed the three assessments (T0, T1 and T2). Twenty children were evaluated by both the clinical observer and the “Observational Monitoring” artificial intelligence system at T0, T1, and T2. At each time point, the six predefined oral-health conditions were recorded by both assessment methods, allowing direct comparison of the observer and AI classifications. The distribution of positive findings across the three assessment times is shown in Figure 1.

### 3.1. Agreement at Baseline (T0)

At baseline (T0), observed agreement between the clinical observer and the AI system ranged from 65.0% for minor food debris to 95.0% for persistent dental calculus, and was 90.0% for satisfactory oral hygiene, 85.0% for mild gingivitis, 80.0% for dental plaque, and 75.0% for dental staining (Table 1). The corresponding 95% CIs were wide, with lower limits ranging from 40.8% (minor food debris) to 75.1% (persistent dental calculus). Chance-corrected agreement was lower and more variable: Cohen’s kappa was substantial for persistent dental calculus (κ = 0.64), moderate for dental plaque (κ = 0.47), and fair for mild gingivitis (κ = 0.32) and minor food debris (κ = 0.30). For satisfactory oral hygiene (κ = 0.00) and dental staining (κ = −0.14), high observed agreement coincided with kappa values close to or below zero; both conditions were recorded in very few children at baseline (0–15%). Minor food debris showed both the lowest observed agreement and low chance-corrected agreement, with five children classified as positive only by the AI system and two only by the observer.

### 3.2. Agreement at 6 Months (T1)

At T1, observed agreement ranged from 65.0% for satisfactory oral hygiene to 100% for persistent dental calculus, which was not recorded in any child by either method, so that kappa could not be computed (Table 2). The 95% CIs for observed agreement were wide, with lower limits ranging from 40.8% (satisfactory oral hygiene) to 83.2% (persistent dental calculus). Agreement was 95.0% for dental staining, 80.0% for dental plaque and minor food debris, and 70.0% for mild gingivitis. Cohen’s kappa was moderate for dental plaque (κ = 0.56) and minor food debris (κ = 0.47), and fair for mild gingivitis (κ = 0.40) and satisfactory oral hygiene (κ = 0.29); these two conditions had the most balanced prevalence at T1 (40–50%). For dental staining, the single discordant classification (one child classified as positive by the AI system only) resulted in κ = 0.00 despite 95.0% observed agreement. Discordant classifications were distributed in both directions, without a systematic tendency of either method to record more positive findings.

### 3.3. Agreement at 12 Months (T2)

At T2, observed agreement ranged from 85.0% for dental staining to 100% for persistent dental calculus and mild gingivitis, which were not recorded in any child by either method, so that kappa could not be computed (Table 3); for these two conditions, observed agreement was 100% (95% CI 83.2–100%). For the remaining conditions, the lower 95% confidence limits ranged from 62.1% (dental staining) to 75.1% (minor food debris). Agreement was 95.0% for minor food debris and 90.0% for dental plaque and satisfactory oral hygiene. Cohen’s kappa was substantial for minor food debris (κ = 0.64) and satisfactory oral hygiene (κ = 0.62), and moderate for dental staining (κ = 0.50) and dental plaque (κ = 0.46).

Although differences between methods were not statistically significant, discordant classifications at T2 tended to follow a consistent direction: all discordant cases for satisfactory oral hygiene (n = 2) and dental staining (n = 3) were recorded as positive by the AI system only, whereas both discordant cases for dental plaque were recorded as positive by the observer only. Given the small sample, these patterns should be regarded as hypothesis-generating.

### 3.4. Changes over Time

The prevalence of the investigated conditions changed markedly during follow-up (Table 4). According to the observer, dental plaque decreased from 70.0% at T0 to 35.0% at T1 and 15.0% at T2 (AI: from 80.0% to 35.0% and 5.0%), and minor food debris decreased from 50.0% to 25.0% and 5.0% (AI: from 65.0% to 25.0% and 10.0%). Satisfactory oral hygiene increased from 0% to 45.0% and 80.0% (AI: from 10.0% to 40.0% and 90.0%). Mild gingivitis increased at T1 (observer 40.0%, AI 50.0%) and was absent at T2, while dental staining, after a decrease at T1, rose to 75.0% according to the observer and 90.0% according to the AI system at T2. Persistent dental calculus was recorded in only one or two children at baseline and in none thereafter. Observed agreement was numerically higher at T2 (85–100%) than at T0 (65–95%); however, this pattern largely reflects the lower prevalence of most findings at T2 and was not formally tested, so it should not be interpreted as an improvement in the performance of the AI system over time.

## 4. Discussion

The present prospective pilot study compared the assessments generated by an AI-assisted remote monitoring system with those obtained during direct clinical examination for six oral health findings in children with mixed dentition. After the observer and AI classifications were analyzed as paired data, observed agreement ranged from 65% to 100% across conditions and time points, whereas chance-corrected agreement was considerably more variable, with Cohen’s kappa values ranging from poor to substantial according to Landis and Koch (−0.14 to 0.64) [17].

At baseline, observed agreement ranged from 65% to 95%, at T1 from 65% to 100%, and at T2 from 85% to 100%. The higher values recorded at T2 should not be interpreted as an improvement of the AI system over time: the three estimates were obtained in a small pilot sample, were not formally compared, and are strongly influenced by the prevalence of each finding, which changed markedly during follow-up. Moreover, with only 20 children, a non-significant McNemar test does not exclude clinically relevant disagreement between the two methods, and a few discordant classifications may substantially change the agreement estimates; accordingly, the 95% CIs for observed agreement were wide, with lower limits as low as 40.8%. Consequently, neither the absence of statistically significant differences nor the high observed agreement should be interpreted as evidence that the two assessment methods are interchangeable.

The discrepancy between observed and chance-corrected agreement deserves specific comment. For satisfactory oral hygiene and dental staining at baseline, and for dental staining at T1, observed agreement ranged from 75% to 95%, whereas kappa was zero or negative. In these cases the condition was recorded in very few children (0–15%), so that the agreement expected by chance was itself very high and one to five discordant classifications were sufficient to produce a kappa close to or below zero. This is the well-known kappa paradox described by Feinstein and Cicchetti [18]. Conversely, for conditions with a more balanced prevalence, such as minor food debris at T0 and mild gingivitis and satisfactory oral hygiene at T1, kappa values (0.29–0.40) were only fair, and observed agreement was also lower (65–70%), indicating genuine disagreement between the two methods. Taken together, these findings suggest that the AI system performed less consistently for findings that require a contextual or global judgement, such as food debris, gingival inflammation and overall oral hygiene, and that observed and chance-corrected agreement should always be interpreted jointly. In addition, the direction of the discordant classifications at T2, with staining and satisfactory oral hygiene recorded more often by the AI system and small plaque deposits recorded more often by the observer, may indicate that remotely acquired images tend to emphasize extrinsic discoloration while underestimating thin plaque layers; this hypothesis should be tested in larger samples.

The variability observed across clinical findings may reflect differences in how easily individual conditions can be recognized from remotely acquired intraoral images. Findings such as plaque deposits or food debris may be influenced by image quality, illumination, camera angulation, and the visibility of dental surfaces, while gingival changes and global judgments of oral hygiene may require a more contextual clinical interpretation. Recent evidence on AI analysis of intraoral photographs similarly indicates that plaque and gingival assessment can be feasible but remains dependent on image standardization and methodological quality [19].

The results should also be interpreted in the context of the distribution of the clinical variables. Several findings were uncommon or absent at specific time points: persistent dental calculus was not recorded in any child at T1 and T2, and mild gingivitis was not recorded at T2, so that agreement for these conditions was 100% by construction and kappa could not be computed. This limitation is particularly relevant in small samples, in which a few discordant classifications may substantially change the agreement estimates. In addition, the prevalence of several findings changed markedly during follow-up (Table 4): dental plaque and minor food debris decreased, satisfactory oral hygiene increased, and dental staining increased at the final assessment. These changes may reflect the oral hygiene instructions and/or preventive care received during follow-up, increased awareness and motivation related to periodic image acquisition at home, and age-related changes during mixed dentition, such as the exfoliation of primary teeth and the eruption of permanent teeth with different surface characteristics. The increase in staining at T2 may be related to dietary habits or the use of chromogenic products. Because both observed agreement and kappa depend on the prevalence of each finding, such shifts in distribution also limit the comparability of agreement estimates across time points.

Remote monitoring has shown potential for orthodontic follow-up, aligner tracking, reduction in in-office visits, and support of oral-hygiene monitoring [4,5,6,7,8,9,10]. Nevertheless, systematic reviews emphasize heterogeneity among available studies and the need for further prospective validation before remote monitoring can be considered equivalent to conventional clinical assessment [11,12]. The present findings are consistent with a cautious interpretation of this technology in younger patients who are not receiving active orthodontic treatment. From a clinical perspective, the Observational Monitoring workflow may still offer advantages as an adjunctive follow-up tool. Periodic image acquisition may provide additional information between conventional visits and may facilitate continuity of observation during mixed dentition. However, the variable chance-corrected agreement observed in the present pilot sample indicates that AI-generated outputs should be reviewed by a dental professional and should not replace direct examination when a clinically relevant change is suspected.

It should be explicitly stated that the present investigation was designed and should be interpreted as a pilot study, and its main limitations are closely linked to this exploratory design. Several limitations should therefore be acknowledged. First, the sample included only 20 children; consistent with the pilot nature of the study, this limited sample size, which was based on feasibility rather than a formal power calculation, reduces statistical power and the precision of the agreement estimates, and findings should therefore be regarded as preliminary. Second, some clinical conditions had very low prevalence or no positive observations at specific time points, which made kappa unstable or not computable for some comparisons and limited the precision of all agreement estimates. Third, the study used a limited set of binary oral-health findings, and the clinical examination, although performed by a calibrated examiner, was carried out by a single observer, and intra-examiner reliability was not formally quantified through repeated assessments (e.g., kappa statistics or intraclass correlation coefficients), as recommended for image-based dental assessments [20]. Fourth, image acquisition at home may introduce variability related to patient or caregiver technique; moreover, the exact interval between home image acquisition and clinical examination was not systematically recorded, and since plaque, food debris and mild gingival inflammation may change within hours, this may have contributed to the observed discordances. Fifth, although the same children were examined three times, the repeated-measures structure of the data was not modelled; with 20 children and sparse positive findings, generalized estimating equations or mixed-effects logistic models would have produced unstable estimates, and agreement at each time point was therefore reported descriptively. Finally, consistent with its pilot design, the study did not evaluate diagnostic accuracy through sensitivity, specificity, predictive values, or receiver-operating-characteristic analysis with the clinical examination as reference standard: because several conditions had few or no positive cases at some time points, such estimates would have been not computable or extremely imprecise.

Building on these preliminary pilot findings, further multicenter prospective studies should include larger and more representative pediatric samples, ideally guided by an a priori sample-size calculation, together with multiple calibrated clinical examiners, standardized image-acquisition protocols, and sufficient numbers of positive and negative cases for each condition, so that diagnostic accuracy (sensitivity and specificity with the clinical examination as reference standard) can also be estimated. The longitudinal structure of the data should be analyzed with generalized estimating equations or mixed-effects logistic models, which allow the effects of the assessment method, time, and their interaction to be estimated while accounting for within-child correlation. Such adequately powered studies will be essential to confirm and further validate the present preliminary results, and to determine which oral findings can be monitored reliably and under which clinical circumstances AI-assisted remote monitoring provides meaningful added value.

## 5. Conclusions

In this prospective pilot study, the AI-assisted Observational Monitoring system showed moderate-to-high observed agreement (65–100%) with direct clinical assessment across six oral-health findings in children with mixed dentition, but chance-corrected agreement was variable and, for several conditions, only fair. For low-prevalence findings, high observed agreement coincided with low kappa values.

These preliminary findings suggest a potential complementary role for AI-assisted remote monitoring as a complementary rather than substitutive approach in pediatric dental follow-up. Professional supervision and clinical verification remain essential, particularly for subtle or infrequent findings. Larger prospective validation studies are required before the system can be considered interchangeable with conventional clinical examination.

## Figures and Tables

**Table 1:** Descriptive analysis of diagnoses made by the observer and the artificial intelligence at baseline (T0), and the degree of agreement.

T0					
	Observer n (%)	Artificial Intelligence n (%)	*p*-Value	Agreement, % (95% CI) *	Κ *
**Dental plaque, n (%)**			0.625		
Present	14 (70.00)	16 (80.00)		80.0 (56.3–94.3)	0.47
Absent	6 (30.00)	4 (20.00)			
**Persistent dental calculus, n (%)**					
Present	1 (5.00)	2 (10.00)	1.00	95.0 (75.1–99.9)	0.64
Absent	19 (95.00)	18 (90.00)			
**Minor food debris, n (%)**					
Present	10 (50.00)	13 (65.00)	0.453	65.0 (40.8–84.6)	0.30
Absent	10 (50.00)	7 (35.00)			
**Mild gingivitis, n (%)**					
Present	3 (15.00)	2 (10.00)	1.00	85.0 (62.1–96.8)	0.32
Absent	17 (85.00)	18 (90.00)			
**Satisfactory oral hygiene, n (%)**					
Present	0 (0.00)	2 (10.00)	0.50	90.0 (68.3–98.8)	0.00
Absent	20 (100.00)	18 (90.00)			
**Dental staining, n (%)**					
Present	3 (15.00)	2 (10.00)	1.00	75.0 (50.9–91.3)	−0.14
Absent	17 (85.00)	18 (90.00)			

*p*-value using the exact McNemar test. * Cohen’s kappa coefficient.

**Table 2:** Descriptive analysis of diagnoses made by the observer and the artificial intelligence at time one (T1), and the degree of agreement.

T1					
	Observer n (%)	Artificial Intelligence n (%)	*p*-Value	Agreement, % (95% CI) *	Κ+
**Dental plaque, n (%)**					
Present	7 (35.00)	7 (35.00)	1.00	80.0 (56.3–94.3)	0.56
Absent	13 (65.00)	13 (65.00)			
**Persistent dental calculus, n (%)**					
Present	0 (0.00)	0 (0.00)	1.00	100.0 (83.2–100.0)	N.C.
Absent	20 (100.00)	20 (100.00)			
**Minor food debris, n (%)**					
Present	5 (25.00)	5 (25.00)	1.00	80.0 (56.3–94.3)	0.47
Absent	15 (75.00)	15 (75.00)			
**Mild gingivitis, n (%)**					
Present	8 (40.00)	10 (50.00)	0.687	70.0 (45.7–88.1)	0.40
Absent	12 (60.00)	10 (50.00)			
**Satisfactory oral hygiene, n (%)**					
Present	9 (45.00)	8 (40.00)	1.00	65.0 (40.8–84.6)	0.29
Absent	11 (55.00)	12 (60.00)			
**Dental staining, n (%)**					
Present	0 (0.00)	1 (5.00)	1.00	95.0 (75.1–99.9)	0.00
Absent	20 (100.00)	19 (95.00)			

*p*-value using the exact McNemar test. * Cohen’s kappa coefficient.

**Table 3:** Descriptive analysis of the diagnoses provided by the observer and the artificial intelligence at the final time point (T2), and the degree of agreement.

T2					
	Observer n (%)	Artificial Intelligence n (%)	*p*-Value	Agreement, % (95% CI) *	Κ *
**Dental plaque, n (%)**					
Present	3 (15.00)	1 (5.00)	0.50	90.0 (68.3–98.8)	0.46
Absent	17 (85.00)	19 (95.00)			
**Persistent dental calculus, n (%)**					
Present	0 (0.00)	0 (0.00)	1.00	100.0 (83.2–100.0)	N.C.
Absent	20 (100.00)	20 (100.00)			
**Minor food debris, n (%)**					
Present	1 (5.00)	2 (10.00)	1.00	95.0 (75.1–99.9)	0.64
Absent	19 (95.00)	18 (90.00)			
**Mild gingivitis, n (%)**					
Present	0 (0.00)	0 (0.00)	1.00	100.0 (83.2–100.0)	N.C.
Absent	20 (100.00)	20 (100.00)			
**Satisfactory oral hygiene, n (%)**					
Present	16 (80.00)	18 (90.00)	0.50	90.0 (68.3–98.8)	0.62
Absent	4 (20.00)	2 (10.00)			
**Dental staining, n (%)**					
Present	15 (75.00)	18 (90.00)	0.250	85.0 (62.1–96.8)	0.50
Absent	5 (25.00)	2 (10.00)			

*p*-value using the exact McNemar test. * Cohen’s kappa coefficient.

**Table 4:** Changes over time in the prevalence of each condition, separately for the observer and the artificial intelligence (T0, T1, and T2).

	T0	T1	T2	*p*-Value
**Dental plaque present, n (%)**				
Observer	14 (70.00)	7 (35.00)	3 (15.00)	0.003
AI	16 (80.00)	7 (35.00)	1 (5.00)	<0.001
**Persistent dental calculus present, n (%)**				
Observer	1 (5.00)	0 (0.00)	0 (0.00)	0.368
AI	2 (10.00)	0 (0.00)	0 (0.00)	0.135
**Minor food debris present, n (%)**				
Observer	10 (50.00)	5 (25.00)	1 (5.00)	0.006
AI	13 (65.00)	5 (25.00)	2 (10.00)	0.002
**Mild gingivitis present, n (%)**				
Observer	3 (15.00)	8 (40.00)	0 (0.00)	0.004
AI	2 (10.00)	10 (50.00)	0 (0.00)	<0.001
**Satisfactory oral hygiene present, n (%)**				
Observer	0 (0.00)	9 (45.00)	16 (80.00)	<0.001
AI	2 (10.00)	8 (40.00)	18 (90.00)	<0.001
**Dental staining present, n (%)**				
Observer	3 (15.00)	0 (0.00)	15 (75.00)	<0.001
AI	2 (10.00)	1 (5.00)	18 (90.00)	<0.001

*p*-value using Cochran’s Q test.

## Data Availability

The data presented in this study are available on reasonable request from the corresponding author. The data are not publicly available due to privacy and ethical restrictions, as they include intraoral images and clinical data of minors.
